# Bibliographical

**Published:** 1879-01

**Authors:** 


### BIBLIOGRAPHICAL.

A treatise on Dental Caries. Experimental and Therapeutic Investigations, by Dr. E. Magetot Lawreate, of the

Institute of France, of the Faculty and the Academy of Medicine, of Paris, etc., etc. Translated by Thomas N. Chandler, D. M. D., Professor of Mechanical Dentistry, and Dean of the Dental School of Harvard University.

This very excellent work has just come to hand, and after a hasty perusal, I am inclined to regard it as the most thorough presentation of the subject of caries of the teeth as yet published. It is the work of a very close observer and accurate experimenter, who presents the leading points of the subject in a very clear, comprehensive and understandable manner. It is also gratifying to see that he either ignores or attaches no material significance, to some mere theories that have been so persistently pressed by some writers, such, for instance, as that leptothrix buccalis and vibriones, are active causes of caries, etc.

Of this work Dr. Chandler says: "No writer has investigated this subject so systematically and thoroughly as Dr. Magetot, and his conclusions are therefore grounded on something more substantial than the speculations which have heretofore formed the basis of our knowledge of the malady. His experiments are definitely stated, and can easily be repeated by any one who doubts their results, while the conclusions logically drawn therefrom must be admitted if we admit his premises." The translator has added foot notes in places, with the view of giving further illustration of some point or points under discussion, and in a few instances, has entered protest against the position of the author, and in each instance, seemingly upon very just ground, at least the advance views and practice of the profession in this country, justify this statement.

The work of the translator seems to be well done, giving a clear and complete presentation of the thoughts and views of the author.

This work should be in the hands of every student and practitioner, both for study and reference.

